# Physical Persistency across Game Quarters and during Consecutive Games in Elite Junior Basketball Players

**DOI:** 10.3390/ijerph19095658

**Published:** 2022-05-06

**Authors:** Rubén Portes, Rafael Manuel Navarro Barragán, Julio Calleja-González, Miguel Ángel Gómez-Ruano, Sergio Lorenzo Jiménez Sáiz

**Affiliations:** 1 Faculty of Sport Sciences, Universidad Europea De Madrid, 28670 Madrid, Spain; sergio.jimenez.saiz@urjc.es; 2 Faculty of Education and Sport, University of the Basque Country (UPV/EHU), 01007 Vitoria, Spain; julio.calleja@ehu.eus; 3 Faculty of Physical Activity and Sport Sciences, Technical University of Madrid, 28040 Madrid, Spain; miguelangel.gomez.ruano@upm.es

**Keywords:** basketball, ratio acceleration, deceleration, women

## Abstract

Given the intermittent nature of basketball and the different demands that occur during playing time that are specific to every level of competition, the ratio of accelerations/decelerations and the intensity level across quarters were evaluated in female elite junior basketball players (n = 48; age: 16.8 ± 0.7 years; height: 1.76 ± 0.07 cm; body mass: 67.2 ± 6.2 kg). The following variables were analyzed to determine physical persistency across game quarters:(a) total distance covered (m), (b) high-intensity running (HIR) (14–21 km·h^−1^) distance covered (m), (c) sprint (21–30 km·h^−1^) distance covered (m), (d) total accelerations (n), (e) total decelerations (n), (f) relative accelerations (n·min^−1^), (g) relative decelerations (n·min^−1^), (h) ratio of acceleration/deceleration (A/D), (i) total jumps (j) relative jumps (n·min^−1^) (k) player load (AU). using the WIMU PRO^®^ system. Higher but shorter acceleration intensity occurred during the last quarters due to the tight results of the matches. The results suggest that high-intensity efforts such as sprints and HIR seem to increase the A/D ratio (guard and forward positions). Therefore, specific conditioning, as well as eccentric strength training, could be included by practitioners in training programs to improve the performance of these positions during competition, especially as a prior preparation to a game-congested event. Centers seem to have a more variable performance through quarters than do other positions, perhaps highlighting the need for specific conditioning strategies.

## 1. Introduction

Basketball is one of the most famous team sports worldwide [1], where optimal performance is highly complex as it requires a combination of technical and tactical abilities and a high degree of physical fitness, among other abilities [2]. Besides, the preparation of this type of players involves developing other physical and psychological attributes [3], given that the games are characterized by repeated explosive activities, such as sprints, jumps, shuffles, and rapid changes of direction [4]. This is the main reason why physical fitness is one of the most important performance factors in basketball [5], playing a “key” role during practice and competitions [6] in a typical congested schedule (a high number of games in a short period of time) [7].

In particular, some studies have evaluated the examined the attributes of female basketball players’ performance [8,9]. In this sense, the periodization plan implemented could improve the physical performance capacity of elite female basketball players [10], with the inclusion of personal physical fitness sessions in order to develop the skills required by each position to improve players’ physical performance [11].

For that reason, a physical fitness profile is dynamically developed over the entire period of growth [12]. The analysis of adolescent basketball players’ capacities is important as it forms the basis for the transition to an established senior category [11]. When analyzing the adolescent players’ performance, the impact of maturation must be accounted for [13]. The development of anthropometrical parameters in U-18 players, including the greatest rate of change in body mass, occurs approximately one year after the growth spurt (peak height velocity). Thus, the time surrounding peak height velocity is considered a potential window of opportunity for strength development [14].

On the other hand, external load represents the variables manipulated to induce internal load [15] and provide an objective assessment of the players’ workload. In that line, IMU’s have been used extensively in the general population as a measure of physical activity level [16]. The monitoring of the external load measurements derived from these triaxial accelerometers is currently considered a valid and reliable tool in team sports [17]. This device allows the recording of data in three planes, reproducing the specific movements performed in basketball, as the combination of defensive and offensive movements forward, backward, and lateral [18]. The type of observed actions includes acceleration and deceleration movements [15].

The current available research evaluated the number of accelerations and decelerations across quarters (first quarter, second quarter, third quarter, and fourth quarter) and playing positions (guard, forward and center). In fact, more intense accelerations were performed in the last quarter, involving faster movements. In addition, guard players performed more accelerations, and their intensity was greater than that of other positions (i.e., forward and center). Particularly, when the acceleration profile was established for the quarters of a basketball game, results were different for guards, forwards and centers in U-18 women’s basketball games, and there was also a lower degree of persistency between quarters [19,20]. Previous studies have found fluctuations in the persistency of demands between quarters in professional male basketball as well [21,22].

The knowledge of the specific persistency of performance during basketball games is a tool of great value for coaches when prescribing training stimulus to analyze persistency (consistency of data registered to analyze performance) of performance across quarters. We studied different variables that were useful to improve this process (Total distance, A/D, and high-intensity distances). Due to the dynamic nature of basketball, the impact of the acceleration/deceleration (A/D) ratio [23] (accelerations minus decelerations as a measure of how efficient in game-specific actions a player is on the court, as well as an indicator of strength deficiencies that could lead to overuse injuries in the lower body) could be very useful to the programing of the training process. This study intends to clarify which variables affect this ratio. Furthermore, this analysis could clarify the impact of the A/D ratio on physical performance during competitive matches [24], and to the best of the authors’ knowledge, no previous scientific evidence has been reported regarding performance persistency among quarters. This is the main reason why this A/D ratio could predict final performance in this population based on positions during congested tournaments. Therefore, the main goal was to analyze the persistency of performance across quarters and games of the A/D ratio in three different playing positions (guard, forward, and center) of women junior basketball players during a game-congested event. Finally, we hypothesize that persistence of performance would decrease in the second or third game of the event and that it would also vary during the quarters of a single game.

## 2. Methods

### 2.1. Subjects

A total of 48 female elite junior basketball players from four different teams (age: 16.8 ± 0.7 years; height: 1.76 ± 0.07 cm; body mass: 67.2 ± 6.2 kg) (Somatic measures obtained with Tanita WB3000, Tanita Company, Tokyo, Japan). The number of players who volunteered to participate in the study broken down by specific position are as follows: guards: n = 22; forwards: n = 13; and centers: n = 13. Players were classified by coaches as guards (point guards and shooting guards), forwards (small forwards and power forwards), and centers. All players were competing in an elite junior competition (the Madrid-Spain Junior Basketball Final Four), which is Madrid´s state tournament, which is played just prior to the Spanish national tournament. All players in this study performed approximately 10 hours of team training (5 total sessions) and six hours of gym-based conditioning per week during the season leading into competition. All players were informed of the aim, risks, and benefits of the study before signing written consent to allow the collection of data for scientific purposes. The study was designed in compliance with the recommendations for clinical research of the Declaration of Helsinki of the World (2008) [25]. 

The local institutional human research ethics committee approved the study protocol (CIPI/18/195).

### 2.2. Observation Period

The competition was played over the course of 3 days (Thursday, Saturday, and Sunday) in the same arena, with 4 female teams playing each of the other female teams. The schedule of the competition for each team is shown in Table 1. Each match consisted of 4 10-minute quarters, with one min separating each quarter and 15 min separating each half (i.e., between quarters 2 and 3). At least 10 min of actual playing time (while the match clock was running) had to be completed in the match being analyzed for player data to be included in the final sample for analysis. Consequently, 133 individual female match samples were included in the final analyses.

### 2.3. Procedures

The external load (EL) was monitored using WIMU PRO^®^ devices (Realtrack Systems SL, Almería, Spain). These devices include an accelerometer, a gyroscope, and a magnetometer sampling at 100 Hz, and they were attached to the upper back of participants during matches with an adjustable harness. The system also uses 6 portable ultra-wideband (UWB) antennae placed within 5 meters of each corner and middle line of the court, collecting positioning data at 20 Hz. The system operates using triangulations between the antennae and the units every 50 Ms. The time required to receive the signal is calculated by the device and the unit position (X, Y, and Z) and derived using one of the antennae as a reference. The antennae remained in the same position across the entire observation period to ensure consistency in the acquired data. The data were analyzed using the WIMU PRO^®^ software (Realtrack Systems SL, Almería, Spain).

#### Dependent Variables

The variables used to indicate EL were: (a) total distance covered (m); (b) high-intensity running (HIR) (14–21 km·h^−1^) distance covered (m); (c) sprint (21–30 km·h^−1^) distance covered (m); (d) total accelerations (n); (e) total decelerations (n); (f) relative accelerations (n·min^−1^); (g) relative decelerations (n·min^−1^); (h) ratio of A/D; (i) total jumps (j); relative jumps (n·min^−1^); (k) player load (arbitrary units (AU) calculated using the following equation: player load n = $\sqrt{[(\mathrm{ACxn}-{\mathrm{ACxn}}^{-1})\;2+\left(\mathrm{ACyn}-{\mathrm{ACyn}}^{-1}\right)\;2+\left(\mathrm{ACzn}-{\mathrm{ACzn}}^{-1}\right)\;2]/100\;}$, where AC(x,y,z) = AC_Body (acceleration minus gravity), ACy is the lateral–medial axis acceleration, ACx is the vertical axis acceleration, ACz is the anteroposterior axis acceleration, and (l) is the relative player load (AU·min^−1^).

The WIMU PRO^®^ has shown adequate reliability to measure team-sport-specific movements [26,27]. Specifically, the UWB (ultra-wideband) technology showed better accuracy (bias: 0.57–5.85%), test–retest reliability (% technical error of measurement (TEM): 1.19), and interunit reliability (bias: 0.18%) in determining distance covered than GPS technology (bias: 0.69–6.05%; %TEM: 1.47; bias: 0.25%) during intermittent, team-sport activity [27]. Also, UWB showed better results (bias: 0.09%; intraclass correlation (ICC): 0.979; bias: 0.01%) in measuring mean movement velocity than GPS technology (bias: 0.18%; ICC: 0.951; bias: 0.03%) during walking (<6 km·h^−1^) and running (>16 km·h^−1^) [27]. The accuracy of the UWB technology has also been tested indoors, showing high sensitivity to relative positioning on the court [28].

### 2.4. Statistical Analysis

The descriptive analysis was conducted using the mean and standard deviation of physical demands according to the game number and game quarter for each playing position. Secondly, the autocorrelation function (ACF), the measurement between the relationship of a variable current value and its past values, was run to calculate the persistency of each physical demand measured within each game (across game quarters) and across the games [29]. This statistical model allows for the defining of the relationships between a series of events (e.g., consecutive games or game quarters within a game). For this analysis, the use of lag 1 was considered to analyze the relationship of each physical demand in each specific game (between games)/game quarter (within game) regarding to the next game/game quarter. This statistical model provides positive or negative (correlation) values that may indicate the performance persistence between and within games for each specific variable. The higher the value, the stronger the persistence in subsequent games/game quarters. All analyses were run using the statistical software IBM SPSS^®^ version 23.0 for Windows (IBM Corp.: Armonk, NY, USA). The statistical significance was established at *p* < 0.05.

## 3. Results

Descriptive results for each physical performance of guards, forwards, and centers during each game quarter for all games are included in Table 2, Table 3 and Table 4, respectively. The results of ACF for guards showed that their physical performance was persistent across the three games (see Table 2) for all variables except the A/D ratio. However, when analyzing the persistency of their performance quarter by quarter in each independent game, the results showed the stable performance of decelerations/min and player load/min during game 1, sprints, accelerations/min, decelerations/min, and jumps/min during game 2, and jumps/min during game 3.

The results of ACF for forwards showed that physical performance was consistent across the three games (see Table 3) for all variables. Their performance was highly consistent quarter by quarter during games 1, 2, and 3 (for all variables except sprints and ratio A/D).

The results of ACF for centers showed that their physical performance was consistent across the three games (see Table 4) for all variables except sprints. However, when analyzing the persistency of their performance quarter by quarter in each independent game, the results showed the stable performance only of total distance and jumps during game 1, accelerations and accelerations/min during game 2, and all variables during game 3.

## 4. Discussion

The main goal was to analyze the persistency of performance across quarters and games and identify the best predictors of the A/D ratio in three different playing positions (guards, forwards, and centers) of women junior basketball players during a game-congested event. The main results of ACF indicated that, when comparing performance across games, the three positions showed similarly persistent performance, with guards showing the highest persistency (A/D ratio), forwards also showing differences in sprint performance, and centers showing lower persistency.

When analyzing performance across quarters in each different game, the results show more differences among positions than when across-game performance was analyzed, as forwards seemed to be the only players performing consistently across both games and quarters in all variables.

The monitoring of the exposure and intensities in competition via IMUs helps in determining performance and fatigue responses, as well as future training strategies [18,21,30,31]. In this regard, the results of our study show variations of activity demands during competition among the different playing positions; this indicates that training programs could be oriented specifically to each playing position although there are general capacities that all positions should develop. Based on the differences of the overall demands among playing positions, it was shown that guards perform a higher number of sprints and undergo more high-intensity shuffling movements compared with forwards and centers [32,33,34,35,36]. This study found differences in the A/D ratio among the guards and the rest of playing positions across games, highlighting the need for specific, eccentric strength training to cope with a higher number of decelerations in this age group. Furthermore, Conte et al. (2015) [37] observed that repeated sprint activity, especially short sprints, is a relevant component of elite women’s basketball. Data collected in this study shows differences in sprint performance across games in centers’ playing positions. According to existing evidence, players in these positions present lower specific physical fitness compared to the other playing positions [11,36]. Our analysis of high-intensity variables over different quarters showed different results than those observed for elite EuroLeague women players [37], partly because of the difference in playing levels, a fact that highlights the specific applicability of results to only the studied age group. Under-18 women’s basketball players present lower persistency in performance over quarters than do professional women basketball players according to the existing literature [20,38]. Evidence found in studies of male basketball players highlighted differences in demands between different playing levels, as Ben Abdelkrim et al. (2010) [39] observed higher decreases in high-intensity activity demands in U−18 male players compared to international players of the same category. The authors attributed these findings to the higher physical fitness of the international players although this study did not investigate the physical capabilities of the participants.

The ability to perform decelerations and changes of direction has shown large correlations to eccentric strength in different studies [40,41,42]. Related to this matter, guards have shown a better-developed capability of generating greater relative strength compared to other playing positions in female basketball [11]. Additionally, elite women basketball back-court players seem to elicit more high-intensity activity and shuffling than do front-court players [36], and guards seem to have lower A/D ratios than do forwards and centers during match play in male basketball [43]. Results for this study showed that, for this particular group and in this competition format, there are also differences between the three studied groups of playing positions, and different demands that are affected by in-game factors that vary depending on specific competition circumstances that highlight the need for analyses to plan and prescribe effective training programs.

This study provides interesting outcomes to the investigation of external load in U-18 women’s basketball players, but there are some limitations encountered in this process. First, the study collected and analyzed only external load data, while comparison of these variables to internal load is recommended to interpret influencing factors of the players performance outcomes [44]. Second, the games were played on consecutive days, which could influence the physical variable outcomes, given the accumulated fatigue in the positions that require lower fitness levels, such as centers [11]; it could also be related to basketball competition factors, such as foul trouble, flow of the game, or playing time. Lastly, more work is required to analyze U-18 women’s basketball players during the season, when games are played once or twice a week; this could affect the periodization of the training suggested in the conclusions, including the same content to cope with training and competition demands, but with adaptable periodization to meet the specific schedule. The conclusions in this study apply only to the studied teams and may not be applicable to players of different playing levels or ages. Future studies could focus on a more holistic approach regarding players’ loads, considering internal and external load in order to control for a more comprehensive approach to the players’ fatigue mechanisms.

## 5. Conclusions

In summary, these results suggest that high-intensity efforts, such as sprints and HIR, seem to affect the A/D ratio (for guard and forward positions). Therefore, specific conditioning, such as repeated sprint ability (RSA), as well as eccentric strength training in the form of eccentric overload, as an evolution of previous general strength training adapted to specific age and gender, could be included by practitioners in training programs to improve the performance of players in these positions in this specific competition format and in this specific age group. In addition, centers seem to have a more variable performance over quarters than do other positions, perhaps highlighting the need for specific conditioning strategies for players in this position in order to maintain high performance throughout the games.

## Figures and Tables

**Table 1:** Match schedule of the tournament.

Time (hh:mm)	Day 1	Day 2	Day 3
10:30		FEMALE 2 vs. 4	FEMALE 1 vs. 4
12:30		FEMALE 1 vs. 3	FEMALE 2 vs. 3
18:30	FEMALE 1 vs. 2		
20:30	FEMALE 3 vs. 4		

**Table 2:** Descriptive statistics for physical demands during games and quarters for guard players (persistency of performances via ACF and *p*-values).

Guards	Game 1										Game 2									
	First		Second		Third		Fourth				First		Second		Third		Fourth			
	M	SD	M	SD	M	SD	M	SD	ACF	*p*	M	SD	M	SD	M	SD	M	SD	ACF	*p*
TDC	409.0	494.7	435.7	420.77	296.2	375.8	394.6	562.7	−0.22	0.53	373.3	393.5	446.3	446.3	380.3	405.6	485.6	409.1	0.17	0.12
HIR	50.78	64.22	47.21	48.99	29.72	37.50	44.82	70.81	−0.24	0.49	44.09	56.50	43.03	48.36	32.19	39.27	33.88	39.95	0.18	0.11
Sprint	5.77	14.03	1.94	3.85	0.36	1.05	1.98	4.22	0.01	0.93	1.49	3.71	5.52	9.26	5.28	11.64	6.12	15.15	0.22	0.04 *
Acc	34.48	42.72	37.13	35.32	32.00	35.76	32.70	46.80	0.03	0.94	51.55	77.90	57.95	60.66	57.70	72.92	71.55	83.33	0.19	0.08
Dec	35.43	42.58	37.35	34.64	31.00	34.48	32.04	46.95	0.04	0.91	32.25	33.24	37.50	36.59	35.10	38.41	46.10	42.05	0.16	0.14
Rel Acc	3 18	3.07	5. 10	3.59	3.88	3.71	2.89	3.49	−0.64	0.07	6.50	6.39	7.89	6.67	7.02	7.17	7.77	7.44	0.32	0.01 *
Rel Dec	3.27	3.03	5.22	3.74	3.71	3.56	3.02	3.37	−0.75	0.03 *	4.88	3.77	4.67	3.27	4.30	3.73	4.89	3.54	0.33	0.01 *
Ratio A/D	−0.48	2.37	−0.35	1.97	−0.48	1.12	−0.74	1.42	−0.08	0.81	−0.90	2.69	0.40	2.14	−0.95	2.91	−1.40	3.07	0.09	0.41
TJ	10.17	14.97	10.43	11.24	8.65	10.46	12.83	18.56	−0.07	0.84	5.40	7.86	5.65	7.16	7.85	8.70	9.00	1204	0.17	0.11
Rel Jumps	0.86	0.93	1.55	1.58	1.05	1.16	1.33	2.11	−0.54	0.13	0.63	0.68	0.55	0.64	0.95	0.93	0.91	0.94	0.34	0.01 *
PL	7.20	8.26	7.47	6.62	5.60	6.46	7.11	9.12	−0.20	0.57	4.94	5.97	5.57	5.48	5.10	5.50	6.29	5.73	0.10	0.36
Rel PL	0.69	0.54	1.10	0.54	0.65	0.59	0.74	0.62	−0.71	0.04 *	0.67	0.55	0.71	0.53	0.64	058	0.67	0.50	0.21	0.06
	**Game 3**										**All Games**									
	**First**		**Second**		**Third**		**Fourth**				**First**		**Second**		**Third**		**Fourth**			
	**M**	**SD**	**M**	**SD**	**M**	**SD**	**M**	**SD**	**ACF**	***p***	**M**	**SD**	**M**	**SD**	**M**	**SD**	**M**	**SD**	**ACF**	***p***
TDC	5691	468.1	510.5	430.6	508.6	499.2	513.7	534.8	0.14	0.21	448.5	456.7	462.8	426.4	390.3	429.8	461.3	503.9	0.24	0.01 *
HIR	5003	58.82	33.02	46.83	29.23	44.71	25.61	36.25	0.13	0.24	48.42	59.25	41.38	47.71	3035	39.82	35.25	52.30	0.23	0.01 *
Sprint	0.85	1.52	0.96	2.70	0.22	0.61	1.71	3.78	−0.03	0.76	2.85	8.94	2.77	6.13	1.88	6.89	3.21	9.22	0.15	0.01 *
Acc	111.75	87.98	104.65	94.22	128.55	134.00	119.00	124.83	0.16	0.14	64.43	77.38	65.17	71.43	70.81	96.31	72.43	94.69	0.30	0.01 *
Dec	49.85	38.86	44.05	41.56	47.95	46.26	48.70	53.08	0.14	0.22	39.00	38.76	39.52	37.08	37.68	39.78	41.79	47.37	0.22	0.01 *
Rel Acc	13.30	7.98	12.61	7.66	11.36	8.66	11.08	9.16	0.20	0.07	7.45	7.31	8.37	6.78	7.25	7.30	7.04	7.67	0.38	0.01 *
Rel Dec	6.11	3.86	5.19	3.23	5.24	4.77	4.51	4.03	0.21	0.05	4.69	3.69	5.03	3.39	4.38	4.02	4.09	3.68	0.29	0.01 *
Ratio A/D	−2.15	2.35	−1.35	2.37	−1.10	2.90	−0.35	3.33	−0.07	0.55	−1.14	2.53	−0.43	2.23	−0.83	2.39	−0.83	2.68	0.11	0.07
TJ	13.60	10.63	12.65	16.74	14.60	15.55	15.20	18.69	0.21	0.05	9.75	12.00	9.62	12.44	10.29	12.04	12.37	16.73	0.25	0.01 *
Rel Jumps	1.72	1.40	1.30	1.20	1.50	1.51	1.46	1.48	0.27	0.01 *	1.06	1.12	1.15	1.28	1.16	1.23	1.24	1.60	0.31	0.01 *
PL	9.63	7.72	8.58	7.61	8.81	8.81	9.04	9.56	0.13	0.25	7.25	7.55	7.22	6.63	6.46	7.11	7.46	8.30	0.22	0.01 *
Rel PL	1.14	0.70	1.06	0.66	0.82	0.62	0.85	0.72	0.17	0.13	0.83	0.63	0.96	0.60	0.70	0.59	0.75	0.61	0.25	0.01 *

* *p* < 0.05; Note: TDC: total distance covered (m); HIR: high-intensity running (m); Sprint: sprint distance covered (m); Acc: total accelerations (n); Dec: total decelerations (n); Rel Acc: relative accelerations (n·min^−1^); Rel Dec: relative decelerations (n·min^−1^); Ratio A/D: ratio of accelerations/decelerations; TJ: total jumps (n); Rel Jumps: relative jumps (n·min^−1^); PL: player load (AU); Rel PL: relative player load (AU·min^−1^).

**Table 3:** Descriptive statistics for physical demands during games and quarters for forward players (persistency of performances via ACF and *p*-values).

Forwards	Game 1										Game 2									
	First		Second		Third		Fourth				First		Second		Third		Fourth			
	M	SD	M	SD	M	SD	M	SD	ACF	*p*	M	SD	M	SD	M	SD	M	SD	ACF	*p*
TDC	746.38	377.98	742.49	431.34	842.25	515.89	875.59	545.20	0.48	0.01 *	598.56	590.57	716.93	485.03	776.60	529.41	527.33	487.58	0.48	0.01 *
HIR	95.00	56.65	85.66	56.88	86.28	61.13	88.50	72.97	0.56	0.01 *	58.49	69.99	51.92	57.73	54.44	61.73	38.29	53.88	0.53	0.01 *
Sprint	3.03	4.78	2.39	4.99	1.00	1.72	5.05	5.74	0.26	0.05	6.53	11.91	2.62	4.44	2.75	4.33	3.05	5.69	0.02	0.85
Acc	64.31	33.49	63.69	34.83	77.46	48.93	74.15	47.69	0.43	0.01 *	78.92	105.41	92.92	85.26	102.92	90.48	64.46	78.26	0.52	0.01 *
Dec	64.54	33.13	62.62	34.87	76.85	49.19	75.23	48.60	0.46	0.01 *	49.31	49.67	64.00	43.74	69.62	47.06	50.31	46.61	0.47	0.01 *
Rel Acc	5.36	2.83	6.37	3.01	5.89	2.83	5.61	2.88	0.42	0.01 *	6.82	6.30	8.37	6.96	8.42	6.71	7.39	5.63	0.63	0.01 *
Rel Dec	5.40	2.64	6.23	2.95	5.56	2.64	5.66	2.83	0.38	0.01 *	4.77	3.38	5.66	3.30	5.69	3.37	5.76	3.51	0.67	0.01 *
Ratio A/D	−0.31	2.81	−0.92	2.53	−0.77	2.42	−0.77	2.83	0.07	0.62	−0.38	2.02	0.00	2.52	−0.15	2.44	−0.15	2.67	0.19	0.16
TJ	18.08	15.79	21.00	18.48	30.23	23.10	27.85	21.81	0.36	0.01 *	11.92	16.57	13.54	12.33	17.23	14.21	12.00	15.13	0.37	0.01 *
Rel Jumps	1.47	1.20	2.18	1.68	2.26	1.55	2.08	1.49	0.30	0.03 *	1.01	1.02	1.26	1.14	1.47	1.13	1.47	1.20	0.44	0.01 *
PL	12.20	6.26	12.22	7.01	14.52	8.70	14.59	9.51	0.40	0.01 *	7.54	8.54	8.68	6.14	9.73	7.00	6.48	6.52	0.45	0.01 *
Rel PL	1.03	0.48	1.27	0.46	1.14	0.58	1.10	0.49	0.27	0.05	0.70	0.53	0.79	0.54	0.80	0.51	0.77	0.52	0.59	0.01 *
	**Game 3**										**All Games**									
	**First**		**Second**		**Third**		**Fourth**				**First**		**Second**		**Third**		**Fourth**			
	**M**	**SD**	**M**	**SD**	**M**	**SD**	**M**	**SD**	**ACF**	***p***	**M**	**SD**	**M**	**SD**	**M**	**SD**	**M**	**SD**	**ACF**	***p***
TDC	609.04	638.58	569.29	514.95	635.98	599.07	573.00	501.28	0.34	0.01 *	651.33	537.30	676.24	471.98	751.61	541.74	658.64	522.30	0.51	0.01 *
HIR	64.60	78.17	66.15	73.99	47.85	58.56	39.45	48.07	0.45	0.01 *	72.70	68.93	67.91	63.24	62.86	61.28	55.41	62.37	0.55	0.01 *
Sprint	3.26	7.29	3.37	9.64	0.03	0.10	2.41	5.72	0.26	0.05	4.27	8.45	2.79	6.60	1.26	2.86	3.51	5.68	0.32	0.01 *
Acc	121.00	127.31	126.00	113.14	166.38	158.56	140.85	121.09	0.37	0.01 *	88.08	97.85	94.21	85.94	115.59	112.76	93.15	92.01	0.47	0.01 *
Dec	51.85	55.54	48.92	42.72	60.38	57.53	56.54	52.66	0.36	0.01 *	55.23	46.32	58.51	40.15	68.95	50.55	60.69	49.22	0.50	0.01 *
Rel Acc	9.64	9.30	11.99	8.40	11.26	9.29	12.33	8.82	0.66	0.01 *	7.27	6.75	8.91	6.78	8.52	7.00	8.44	6.75	0.58	0.01 *
Rel Dec	4.10	4.01	4.83	3.62	5.15	5.42	4.81	3.69	0.48	0.01 *	4.76	3.35	5.57	3.27	5.47	3.89	5.41	3.31	0.53	0.01 *
Ratio A/D	0.38	2.90	−0.08	1.12	−0.69	1.75	0.15	1.28	−0.02	0.90	−0.10	2.56	−0.33	2.14	−0.54	2.19	−0.26	2.34	0.14	0.08 *
TJ	13.92	15.68	15.08	14.96	16.62	19.29	18.62	22.67	0.33	0.01 *	14.64	15.81	16.54	15.40	21.36	19.75	19.49	20.69	0.42	0.01 *
Rel Jumps	1.05	1.09	1.41	1.11	1.10	1.08	1.54	1.71	0.52	0.01 *	1.18	1.09	1.62	1.36	1.61	1.33	1.70	1.47	0.43	0.01 *
PL	9.76	10.30	9.64	8.83	10.37	9.44	9.84	8.47	0.34	0.01 *	9.83	8.52	10.18	7.37	11.54	8.49	10.30	8.72	0.46	0.01 *
Rel PL	0.78	0.75	0.90	0.65	0.73	0.63	0.86	0.64	0.65	0.01 *	0.84	0.60	0.99	0.58	0.89	0.59	0.91	0.56	0.54	0.01 *

* *p* < 0.05; Note: TDC: total distance covered (m); HIR: high-intensity running (m); Sprint: sprint distance covered (m); Acc: total accelerations (n); Dec: total decelerations (n); Rel Acc: relative accelerations (n·min^−1^); Rel Dec: relative decelerations (n·min^−1^); Ratio A/D: ratio of accelerations/decelerations; TJ: total jumps (n); Rel Jumps: relative jumps (n·min^−1^); PL: player load (AU); Rel PL: relative player load (AU·min^−1^).

**Table 4:** Descriptive statistics for physical demands during games and quarters for center players (persistency of performances via ACF and *p*-values).

Centers	Game 1										Game 2									
	First		Second		Third		Fourth				First		Second		Third		Fourth			
	M	SD	M	SD	M	SD	M	SD	ACF	*p*	M	SD	M	SD	M	SD	M	SD	ACF	*p*
TDC	466.58	391.25	568.87	406.67	486.91	408.42	466.16	359.29	0.29	0.04 *	330.39	463.54	442.73	347.73	422.59	376.31	445.35	458.60	0.07	0.62
HIR	66.86	53.69	81.95	69.97	52.96	53.94	58.29	48.88	0.17	0.22	44.27	75.64	35.96	42.43	37.06	47.20	29.76	43.58	0.01	0.93
Sprint	5.14	12.91	2.76	3.96	2.11	7.30	9.71	31.87	−0.04	0.79	1.59	2.34	4.94	10.73	3.99	9.24	3.16	5.79	0.07	0.59
Acc	41.08	32.12	48.00	30.55	37.00	35.99	43.00	34.40	0.18	0.19	43.31	78.80	50.69	47.50	44.38	43.63	55.38	74.55	0.28	0.04 *
Dec	41.58	33.50	48.25	31.76	35.83	34.11	43.08	34.32	0.18	0.19	26.85	39.13	39.62	29.25	37.15	33.99	38.62	36.33	0.05	0.70
Rel Acc	4.48	2.71	5.60	2.75	4.25	3.34	5.73	2.97	0.26	0.06	6.23	5.58	6.67	4.99	5.66	4.88	5.30	5.33	0.34	0.01 *
Rel Dec	4.56	2.67	5.56	2.88	4.19	3.24	5.68	2.82	0.28	0.05	6.29	7.99	5.34	3.16	4.73	3.41	4.07	3.50	0.13	0.32
Ratio A/D	−0.08	1.38	0.08	1.68	0.75	1.22	0.58	0.67	0.05	0.75	−0.54	1.27	1.08	1.98	−0.38	0.65	−0.31	2.29	0.07	0.58
TJ	15.17	13.33	21.83	17.83	17.50	18.17	21.83	21.88	0.29	0.04 *	5.62	9.66	8.23	7.13	8.31	9.44	9.92	10.82	0.20	0.14
Rel Jumps	1.59	1.24	2.60	1.93	2.04	1.93	2.72	1.98	0.26	0.06	0.61	0.71	1.08	0.77	1.01	1.03	1.18	1.52	0.19	0.15
PL	7.88	6.48	9.56	6.43	7.02	6.67	8.32	6.57	0.19	0.18	4.13	6.36	5.19	3.97	4.85	4.30	5.38	6.09	0.13	0.32
Rel PL	0.84	0.52	1.13	0.46	0.82	0.64	1.16	0.50	0.21	0.12	0.51	0.48	0.70	0.40	0.62	0.47	0.53	0.47	0.22	0.10
	**Game 3**										**All Games**									
	**First**		**Second**		**Third**		**Fourth**				**First**		**Second**		**Third**		**Fourth**			
	**M**	**SD**	**M**	**SD**	**M**	**SD**	**M**	**SD**	**ACF**	***p***	**M**	**SD**	**M**	**SD**	**M**	**SD**	**M**	**SD**	**ACF**	***p***
TDC	392.28	444.93	528.59	516.41	427.18	506.45	405.19	475.68	0.55	0.01 *	394.57	427.24	511.94	421.55	444.47	423.75	438.18	425.00	0.42	0.01 *
HIR	38.35	53.17	57.06	59.06	36.10	58.25	29.78	39.64	0.45	0.01 *	49.38	61.48	57.70	59.40	41.75	52.42	38.77	44.90	0.29	0.01 *
Sprint	2.65	5.13	2.43	4.52	0.06	0.18	1.40	5.05	0.31	0.02 *	3.08	7.88	3.39	7.07	2.05	6.80	4.62	18.27	0.04	0.59
Acc	83.77	96.81	114.54	116.21	99.85	119.87	98.85	115.19	0.55	0.01 *	56.45	75.89	71.68	79.82	61.03	80.48	66.34	83.95	0.56	0.01 *
Dec	32.92	37.00	43.00	40.61	34.46	41.50	33.77	37.83	0.55	0.01 *	33.58	36.21	43.50	33.54	35.82	35.78	38.37	35.45	0.38	0.01 *
Rel Acc	9.84	9.52	10.30	8.56	7.62	8.59	9.50	9.20	0.47	0.01 *	6.91	6.84	7.57	6.19	5.89	6.07	6.87	6.56	0.46	0.01 *
Rel Dec	4.02	3.95	4.07	3.41	2.65	3.02	3.35	3.32	0.41	0.01 *	4.96	5.37	4.97	3.15	3.85	3.26	4.33	3.30	0.30	0.01 *
Ratio A/D	−1.46	2.30	−0.69	2.29	−0.15	0.38	−0.46	0.97	0.41	0.01 *	−0.71	1.77	0.16	2.09	0.05	0.93	−0.08	1.53	0.25	0.01 *
TJ	14.46	18.90	22.85	28.35	16.08	20.60	13.00	15.04	0.49	0.01 *	11.66	14.78	17.53	20.45	13.87	16.78	14.74	16.71	0.45	0.01 *
Rel Jumps	1.85	2.20	2.20	2.35	1.28	1.67	1.33	1.52	0.47	0.01 *	1.34	1.58	1.94	1.87	1.42	1.60	1.72	1.77	0.49	0.01 *
PL	6.67	7.61	9.62	9.15	7.15	8.51	6.73	7.79	0.56	0.01 *	6.18	6.84	8.09	7.00	6.32	6.62	6.77	6.78	0.44	0.01 *
Rel PL	0.83	0.85	0.93	0.82	0.55	0.63	0.66	0.65	0.41	0.01 *	0.72	0.64	0.91	0.60	0.66	0.57	0.77	0.60	0.41	0.01 *

* *p* < 0.05; Note: TDC: total distance covered (m); HIR: high-intensity running (m); Sprint: sprint distance covered (m); Acc: total accelerations (n); Dec: total decelerations (n); Rel Acc: relative accelerations (n·min^−1^); Rel Dec: relative decelerations (n·min^−1^); Ratio A/D: ratio of accelerations/decelerations; TJ: total jumps (n); Rel Jumps: relative jumps (n·min^−1^); PL: player load (AU); Rel PL: relative player load (AU·min^−1^).
